# Linking atrial fibrillation with non-alcoholic fatty liver disease: potential common therapeutic targets

**DOI:** 10.18632/oncotarget.19522

**Published:** 2017-07-24

**Authors:** Ya-Hui Ding, Yuan Ma, Lin-Yan Qian, Qiang Xu, Li-Hong Wang, Dong-Sheng Huang, Hai Zou

**Affiliations:** ^1^ Department of Cardiology, Zhejiang Provincial People's Hospital, Hangzhou 310014, China; ^2^ Department of Hepatobiliary Surgery, Zhejiang Provincial People's Hospital, Hangzhou 310014, China; ^3^ People's Hospital of Hangzhou Medical College, Hangzhou 310014, Zhejiang Province, China

**Keywords:** non-alcoholic fatty liver disease, atrial fibrillation, adiponectin, insulin resistance, renin angiotensin aldosterone system

## Abstract

Non-alcoholic fatty liver disease (NAFLD) and atrial fibrillation (AF) are common chronic non-infectious diseases with rising incidences. NAFLD is an independent risk factor for the onset of AF, after adjusting potentially related factors. The pathogenesis of these diseases share several mechanisms including reduced adiponectin level, insulin resistance, and renin angiotensin aldosterone system (RAAS) activation, in addition to activation of common disease pathways that promote inflammation, oxidative stress, and fibrosis. Furthermore, statins and RAAS blockers exert therapeutic effects concurrently on NAFLD and AF. The common pathogenesis of NAFLD and AF may serve as a potential therapeutic target in the future.

## INTRODUCTION

Non-alcoholic fatty liver disease (NAFLD) is the most common chronic liver disease worldwide with growing incidence [1]. It is defined by hepatic fat accumulation and hepatic steatosis resulting from factors other than excessive alcohol consumption. There are two forms of NALFD: non-alcoholic fatty liver (NAFL) and non-alcoholic steatohepatitis (NASH) [2]. The incidence of NAFLD varies between studies from 6.3 to 33% (median 20%) due to the diversity of study populations and diagnostic tools used in each study [1]. The incidence of NASH is relatively low, ranging between 3 and 5%. About 30–40% of NAFL cases progress into fatty liver cirrhosis in 10 years, making NAFLD one of the most common cause of cryptogenic cirrhosis [3]. NAFLD is also considered to be part of multi-organ diseases associated with several other diseases including cardiovascular diseases (CVDs), diabetes, chronic kidney disease, and colorectal cancer [4, 5].

Atrial fibrillation (AF), a common arrhythmia, is characterized by chaotic atrial activation accompanied by atrial dynamics degradation. AF complications include thromboembolism, heart failure, and a reduced quality of life [6]. In the 2010 Global Burden of Disease Study, the age-adjusted prevalence of AF worldwide was reported at 5.96 per 1,000 in men and 3.73 per 1,000 in women. The total number of AF cases is estimated at about 33 million [7], with a growing incidence in recent years. After adjusting for age and sex, Miyasaka et al. reported an AF incidence between 3.04 per 1,000 people in 1980 to 3.68 per 1,000 people in 2000 [8], with a relative increase of 12.6% over 21 years. The growing incidence of AF is associated with several factors including age, male sex, hypertension, valvar heart disease, chronic heart failure, and thyroid disease.

Recent clinical investigations revealed a significant increasing in AF cases among patients with NAFLD, suggesting that NAFLD is an independent risk factor for AF [9–13]. This article reviews the current literature about the epidemiology and mechanisms of NAFLD and AF, and discusses the relationship between the two conditions with the aim to provide new insights into the treatment of both NAFLD and AF.

### NAFLD and AF in epidemiology

The incidence of AF is significantly higher in patients with NAFLD compared to patients without NAFLD after adjusting for potentially related factors such as age, sex, blood pressure, and blood glucose level.

In a 10-year follow-up study of 400 patients with type-2 diabetes, Targher et al. found that NAFLD was closely related to a growing risk of incidental AF (odds ratio [OR] 4.49, 95% confidence interval [CI] 1.6–12.9, *p* < 0.005). The adjusted-OR was 6.38 (95% CI 1.7–24.2, *p* = 0.005) after adjusting for age, sex, hypertension, and electrocardiographic features (left ventricular hypertrophy and PR interval) [12]. Another cross-sectional study from the same group showed a similar result in 702 patients with type-2 diabetes (OR 3.04, 95% CI 1.54–6.02, *p* < 0.001) [13]. The risk for AF in patients with NAFLD decreased when adjustments were made for age, sex, systolic blood pressure, glycated hemoglobin HbA1c, estimated glomerular filtration rate, total cholesterol, electrocardiographic left ventricular hypertrophy, chronic obstructive pulmonary disease, and prior history of heart failure, valvar heart disease, or hyperthyroidism (adjusted-OR 5.88, 95% CI 2.72–12.7, *p* < 0.001). Furthermore, a 16.3-year study of 958 middle-aged patients with hypertension from the Oulu Project Elucidating Risk of Atherosclerosis (OPERA) cohort revealed that NAFLD was associated with an increased risk of AF (Hazard ratio 1.96, 95% CI 1.29–2.97) [10]. 14.9% of patients with NAFLD were diagnosed with AF, while only 7.9% of patients without NAFLD had AF. Similarly, an OR of 1.88 (95% CI, 1.03–3.45) was demonstrated after adjusting for age, sex, study group, diabetes, body mass index, waist circumference, alcohol consumption, smoking, serum alanine aminotransferase concentration, systolic blood pressure, quick index, left ventricular mass index, left atrial diameter, coronary artery disease, atrial natriuretic peptide, and high sensitive C-reactive protein (hsCRP).

A 12 year follow-up of aspartate aminotransferase (AST) and alanine aminotransferase (ALT) levels in 9,333 men and women found a U-shaped association between AF risk and AST/ALT in participating in the Atherosclerosis Risk in Communities Study [14]. The association weakened after adjusting for potential confounders. 3,744 participants without clinical heart failure showed that AST and ALT levels were significantly associated with an increased risk of incidental AF (ALT hazard ratio 1.19, 95% CI 1.07–1.32, *p* = 0.002; AST hazard ratio 1.12, 95% CI 1.01–1.24, *p* = 0.03) in the Framingham Heart Study Original and Offspring cohorts. The associations remained after excluding moderate-to-severe alcohol consumption [15]. Because circulating levels of liver enzymes, like AST, ALT and gamma glutamyl transpeptidase (GGT), are often important markers of NAFLD. The above two studies suggest that NAFLD could be a predictor of AF [16].

Therefore, regardless of whether patients have type-2 diabetes or hypertension, NAFLD remains a significant independent risk factor of AF, particularly in patients with type-2 diabetes (Table 1). Structural and electrical remodeling of atrium induced by inflammation, insulin resistance, lipid metabolism disorder, and fibrosis may play an important role in NAFLD patients, but unfortunately, underlying mechanisms of linking AF with NAFLD is unclear [17].

**Table 1:** The relevance of NAFLD and AF in epidemiology

Subjects	Study protocol	Odds ratio/Hazard ratio	Reference
Type 2 diabetes patients, *n* = 400	Cohort study, follow-up 10 years	4.49 [95% CI 1.6–12.9] adjusted-OR* 6.38 [95% CI 1.7–24.2]	Giovanni Targher^12^
Type 2 diabetes patients, *n* = 702	Cross-sectional study	3.04 [95% CI 1.54–6.02] adjusted-OR 5.88 ** [95% CI 2.72–12.7]	Giovanni Targher^13^
Middle-aged hypertensive patients, *n* = 958	Cohort study, mean follow-up 16.3 years (median 17.6 years, range 0–19 years)	Hazard ratio 1.96 (95% CI) 1.29–2.97 Adjusted OR*** 1.88 [95% CI 1.03–3.45]	Aki J. Käräjämäki^10^

*Adjustments for age, sex, hypertension and electrocardiographic features (left ventricular hypertrophy and PR interval).

**Adjustments for age, sex, systolic BP (blood pressure), HbA1c, (glycated haemoglobin), estimated GFR (glomerular filtration rate), total cholesterol, electrocardiographic LVH (left ventricular hypertrophy), COPD (chronic obstructive pulmonary disease), and prior history of HF (heart failure), VHD (valvular heart disease) or hyperthyroidism.

***age, sex, study group, diabetes, body mass index (BMI), waist circumference, alcohol consumption, smoking, serum alanine aminotransferase concentration (ALT), systolic blood pressure, quick index, left ventricular mass index, left atrial diameter, coronary artery disease (CAD), atrial natriuretic peptide (ANP) and high sensitive C-reactive protein (hs-CRP).

### The mechanism linking AF with NAFLD

Although the above-mentioned epidemiologic investigations revealed that NAFLD is closely related to the incidence of AF, there is still a lack of direct evidence of a correlation between NAFLD and AF pathogenesis. Potential mechanisms involve insulin resistance, oxidative stress, and inflammation

### The role of adiponectin in the pathogenesis of NAFLD and AF

**Figure 1:**
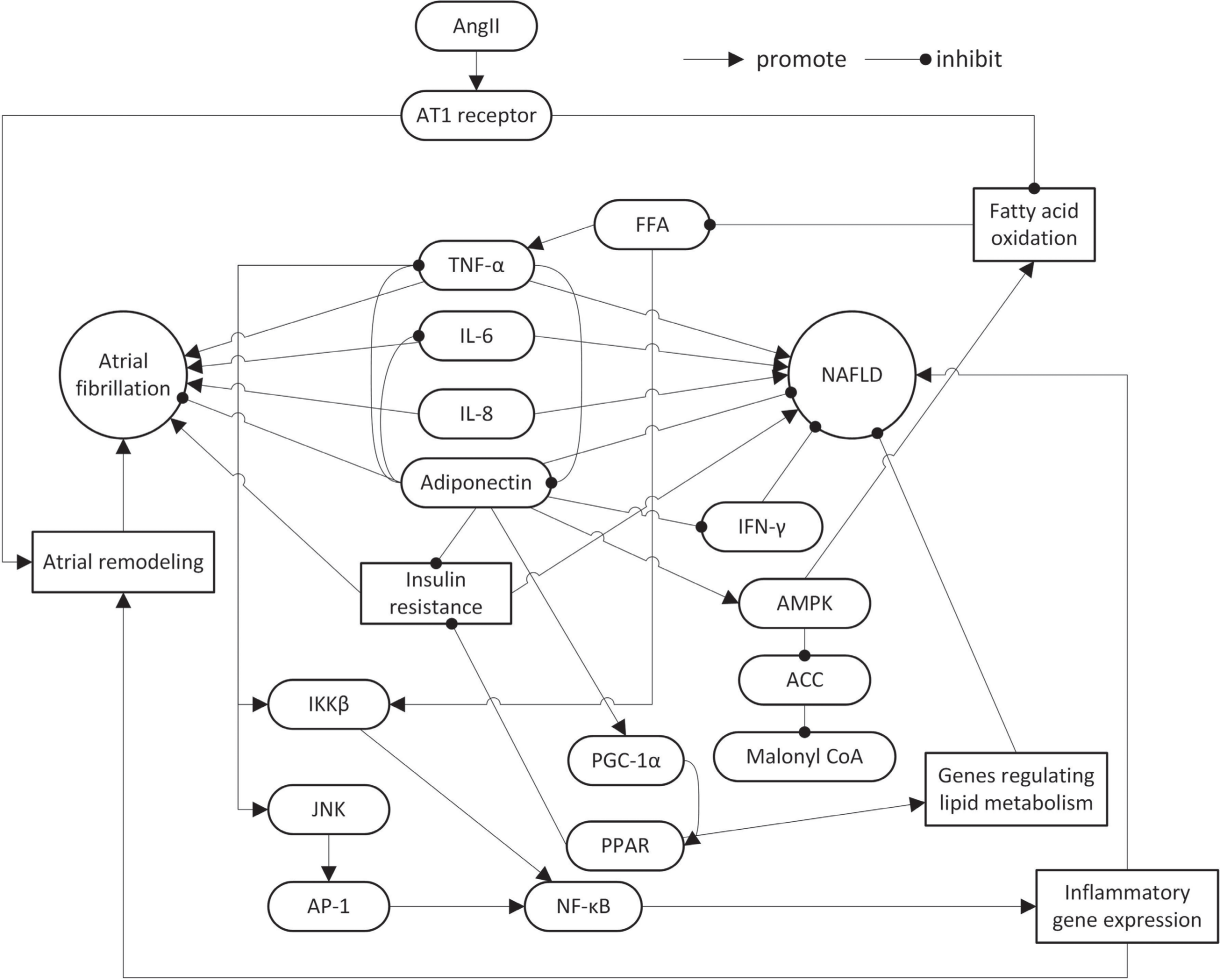
The roles of adiponectin, inflammatory cytokines, AngII and insulin resistance in the pathogenesis of NAFLD and AF NAFLD: non-alcoholic fatty liver disease; AngII: angiotensin II; AT1: angiotensin II type 1; TNF-α: tumor necrosis factor-α; IL: interleukin; IFN-γ: interferon-γ; FFA: free fat acid; AMPK: adenosine monophosphate-activated protein kinase; ACC: acetyl-CoA carboxylase; IKK: inhibitor of nuclear factor kappa-B kinase; JNK: c-Jun N-terminal kinase; AP-1: Activator Protein-1; PPAR: peroxisome proliferator-activated receptor; PGC-1: PPAR γ coactivator-1; NF-κB: nuclear factor κ-light-chain-enhancer of activated B cells.

Adiponectin is an adipokine secreted by adipocytes that is inversely correlated with body fat. Adiponectin significantly impacts the cellular metabolism of glucose and fatty acids, and has several other properties including anti-inflammation, anti-oxidation, anti-atherosclerosis functions and improving insulin resistance (IR) [18, 19]. Parasecretion of adiponectin is associated with hypertension, atherosclerosis, NAFLD, AF, cancer, and other diseases [20]. Inflammatory cells infiltrating adipose tissue secrete cytokines, such as tumor necrosis factor-α (TNF-α), interleukin-6 (IL-6), IL-8, IL-18, and monocyte chemotactic protein-1 (MCP-1), to regulate glucose metabolism, lipid metabolism, and inflammatory response with adiponectin in insulin-sensitive tissues. The imbalance between adipokines and cytokines contributes to the development of IR and NAFLD [21–23]. Furthermore, Hui et al. reported that high TNF-α levels and hypoadiponectinemia are IR-independent features of NASH [24] (Figure 1).

Adiponectin exerts its effects in the liver via AdipoR2, a highly liver-specific adiponectin receptor expressed on the surface of hepatic cells [25]. According to a meta-analysis of serum adiponectin from patients with NAFLD, which analyzed 27 studies with a total of 2,243 participants (including 698 controls and 1,545 patients), a higher serum adiponectin concentration was observed in controls compared to patients with NAFLD and NASH. Furthermore, the serum adiponectin concentration was higher in patients with NAFLD compared with patients with NASH [26]. The reported differences were observed across the body mass index, age, and sex, and in addition to type-2 diabetes mellitus. Interestingly, in the studies where liver biopsies had been performed in controls, there was no significant difference between controls and patients. Another systematic review included four studies, which involved in 187 histologically confirmed NASH adult patients, revealed that Thiazolidinediones treatment increases circulating adiponectin levels as well as histological improvement in NASH patients [27]. Alisol A 24-acetate was also found to improve NASH likely through adiponectin [28]. Hong et al. found that regular supplementation of soybean embryos might prevent NAFLD through adiponectin-mediated AMPKα pathway [29]. Therefore, adiponectin is regarded as an important role for improvement of NAFLD.

Recent studies also reported a correlation between adiponectin and AF. Inflammation and oxidative stress cause atrial remodeling with increasing serum TNF-α level, which is positively correlated with the dilation of the left atrium [30, 31]. Adiponectin's anti-inflammatory and anti-oxidative properties could potentially improve atrial remodeling. Therefore, the serum adiponectin level may be an indicator for evaluating atrial remodeling in patients with AF [32]. Using eicosapentenoic acid in a rabbit model reduced the incidence of AF by increasing adiponectin concentration and decreasing TNF-α concentration in the atrium tissue and the surrounding adipose tissue [33]. After assessing 90 post-operation patients who underwent cardiac surgery, Kourliouros et al. found that post-operation AF is independent of serum IL-6, serum adiponectin, and epicardial IL-6, but is associated with a significant reduction of epicardial adiponectin [34]. Therefore, epicardial adiponectin has a potential protective effect against post-operation AF. Another study in post-operative coronary artery bypass patients showed that hypoadiponectinemia leads to an increased incidence of obesity, metabolic syndrome, and post-operation AF [35]. Notably, Thiazolidinediones showed a significant reduction of AF in type 2 diabetes, but it is unclear if adiponectin is involved [36, 37]. However, some studies found higher adiponectin levels might be associated with a greater risk for AF in older adults [38–40].

The reduction of adiponectin level is a potential common mechanism of NAFLD and AF. However, the correlation between adiponectin and AF, contradicts adiponectin's inhibition of NAFLD and AF genesis and development. Additional work is necessary to define the role of adiponectin in AF.

### The role of insulin resistance in the pathogenesis of NAFLD and AF

Insulin resistance (IR) is the low response of tissue cells (i.e. muscle, adipose tissue, and liver) to circulating insulin. IR primarily results from inefficient insulin uptake and use, and compensatory hyperinsulinemia [41, 42].

IR promotes NAFLD by accelerating NAFLD development and transforming NAFL into NASH [43–45]. Approximately 70% of patients with type-2 diabetes suffer from NAFLD [46, 47]. Liver fat is closely correlated with the ability of insulin to suppress lipolysis. In patients with IR, lipase activity is enhanced and increases long-chain fatty acids (LCFA) in portal blood, which results in more LCFA for hepatic cells, thus worsening NAFLD [48]. In fact, in “Obese/Metabolic NAFLD”, NAFLD is closely associated with IR. However NAFLD caused by genetic variants, like patatin-like phospholipase domain-containing 3 I148M gene variant, are not associated with IR [49].

IR is also a risk factor for AF pathogenesis [50]. IR enhances pro-inflammatory responses in myocardium cells via IL-1, CRP, and reactive oxygen species (ROS). This results in myocardium remodeling, atrium enlargement, and autonomic neuropathy, and may thus promote the occurrence of AF [51–54]. Bissinger and colleagues reported that diabetes mellitus led to autonomic neuropathy and increased the duration and dispersion of P wave [51, 55]. Wang et al. also found that P-wave dispersion and maximum duration are independently associated with IR in patients with metabolic syndrome [56], suggesting that IR contributes to AF development. However, analysis of 3,023 middle-aged to elderly participants from the Framingham Heart Study found that IR does not correlated with incident AF (hazard ratio 1.18, 95% CI 0.84–1.65, *p* = 0.34) [57].

It is worth noting that the mechanisms of IR acting on NAFLD and AF are not the same. While increased LCFA plays a crucial role in NAFLD, it is the IR-induced enhancement of inflammation in myocardium and autonomic neuropathy that may be the major cause of AF.

### The role of renin angiotensin aldosterone system in the pathogenesis of NAFLD and AF

Early studies of the renin angiotensin aldosterone system (RAAS) mostly focused on its role in hypertension. However, recently, the role of RAAS in other diseases has attracted great attention. In addition to its regulatory effect on blood pressure, RAAS promotes the organization of fibrosis in some tissues. Angiotensin II (AngII), an important component of RAAS and a pro-inflammatory factor, upregulates cytokines, promotes cell proliferation, and regulates extracellular matrix metabolism via the AngII type I (AT1) receptor [58]. In humans, the AT1 receptor is mainly distributed in blood vessels, the heart, the liver, the brain, the lung, the kidney, and the adrenal cortex; thus, it may be involved in the genesis and development of NAFLD and AF.

RAAS is an important lipid metabolism signaling pathway in the liver of patients with NAFLD. In rodent models, the blockade of RAAS attenuates obesity caused by high fat diets [59, 60]. Furthermore, knockout rodent models lacking RAAS-related genes such as renin, angiotensin converting enzyme, or bearing liver-specific deletion of AT1 receptor show improved hepatic steatosis [59, 61, 62]. Angiotensin converting enzyme inhibitor and angiotensin receptor blocker have preventive and therapeutic effects against triacylglycerol (TAG) accumulation in the liver [63–67]. This may be associated with the role of AngII in accentuating TAG accumulation in the liver. Its mechanisms involve altering serum free fatty acid and TAG, reducing fatty acid oxidation, regulating secretion of very low density lipoprotein (VLDL), and increasing fatty regeneration. Moreover, AngII suppresses insulin functions by increasing oxidative stress, thus resulting in an increased level of circulating free fatty acid [60, 68, 69]. The activation of RAAS down-regulates the expression of genes related to fatty acid oxidation to suppress the oxidation metabolism of fatty acid [59, 61, 70]. In addition, some cytokines (TNF-α, MCP-1, and IL-6) and IR participate in the process of RAAS-related aggravation of NAFLD.

Angiotensin-converting enzyme inhibitors (ACEi) and AngII receptor blocker (ARB) reduce the risk of developing AF according to retrospective studies from large randomized controlled trials. These findings suggests that the RAAS contributes to the incidence of AF. However, two other trials concluded that upstream RAAS inhibition couldn't increase benefit for AF prevention [71]. Activation of RAAS is an important cause of myocardial fibrosis, and AngII plays a key role in the signaling pathway [72–74]. Myocardial fibrosis causes both electrical and mechanical remodeling of the left atrium, resulting in a higher risk of AF [75–78].

### Effects of drugs on NAFLD and AF

To date, there are no specific drugs that treat both NAFLD and AF. However, several treatments have been reported to exert therapeutic actions on both NAFLD and AF. Among these, statins and RAAS blockers are the most frequently prescribed.

### Role of statin in the treatment of NAFLD and AF

Statins are cholesterol-lowering drugs that are mainly used to treat atherosclerosis. In recent years, the potential therapeutic effect of statins in NAFLD and AF has been postulated.

Considering the high cardiovascular risk in most patients with NAFLD, the use of statins became a reasonable choice in these cases. Despite previous concerns, recent evidence has confirmed the safety of statins and their effects on reducing alanine aminotransferase levels in the liver and the risk of cardiovascular diseases [79, 80]. Indeed, three large randomized controlled studies found that atorvastatin, pravastatin, and pitavastatin could decrease the level of alanine aminotransferase in patients with NAFLD [81–83]. Based on these results, the use of statins to treat patients with NAFLD has been accepted by the standard guidelines [2, 84].

Statins improve NAFLD via multiple pathways. Animal studies have shown that statins prevent the development of inflammation and fibrosis in the liver. Their mechanisms include attenuating the activity of stress-activated c-Jun N-terminal kinase (c-JNK), decreasing the expression of transforming growth factor-β (TGF-β) and connective tissue growth factor, improving peroxisomal β-oxidation, inhibiting the activation of hepatic stellate cells, and up-regulating endothelial nitric oxide synthase and induced nitric oxide synthase [85–87]. These effects are mediated by anti-inflammation, anti-apoptosis, and anti-oxidation. However, due to the lack of histologic evidence, the absence of pathogenesis, and small sample size in currently available studies, the reported results have been inconsistent. Some studies showed that statin treatment reduced hepatic steatosis in the liver [88–91], while other studies did not [92, 93]. Moreover, improved inflammation was only reported in some of these studies [89–91].

Statins help reduce the incidence of post-operation AF in patients after cardiac surgery [94–97]. A similar effect can be found in patients with end-stage renal disease [98]. A study in Asian patients with AF who were treated with amiodarone showed that atorvastatin significantly reduced the incidence of AF relapse and the level of serum hsCRP [99]. However, another study with patients over 70 years of age undergoing coronary artery bypass grafting showed that statin treatment did not reduce the incidence of AF and mortality [100]. Furthermore, Zheng et al. [80] found that, in 1,922 elective patients after cardiac surgery, the administration of 20 mg rosuvastatin did not attenuate the incidence of AF and cardiac injury, but likely increased the risk of acute kidney injury [101].

However, these results have the risks of discrepancy and bias because they are based on retrospective or observational studies. Therefore, more prospective randomized controlled studies are needed to clarify the role of statin in preventing AF. The mechanism of statins in AF prevention remains vague, but may relate to inflammation and oxidative stress. Serum levels of hsCRP, IL-6, and TNF-α increase significantly in AF and can be decreased by statin [97, 99, 102, 103]. Zakkar et al. [83] also found that oxidative stress and inflammation are important factors in post-operative AF in patients following cardiac surgery [104]. In addition, statins increase the serum level of adiponectin, which is another probable reason for decreasing the incidence of AF and NAFLD [105, 106].

### Role of RAAS blockers in the treatment of NAFLD and AF

RAAS plays a crucial role in the pathogenesis of NAFLD and AF. Previous studies found that using angiotensin receptor blockers or decreasing the level of AngII can increase adiponectin. Therefore, RAAS blockers may be a potential therapeutic for treating NAFLD and AF [63, 66, 67].

Furthermore, RAAS blockers use other mechanisms for treating NAFLD and AF. For example, losartan was found to suppress the development of liver fibrosis in rats by inhibiting the expression of the Toll-like receptor (TLR4) and nuclear factor ‘kappa-light-chain-enhancer’ of activated B-cells (NF-κB), as well as attenuating lipopolysaccharide-induced increase in myeloid differentiation factor 88, NF-κB, and TGF-β [107]. In rats with type-2 diabetes, Qiang et al. demonstrated that valsartan could block the pathological course of liver fibrosis by down-regulating the expression of α-smooth muscle actin, TGF-β1, TNF-α, and MCP-1, and anti-apoptosis to restore the injured hepatic mitochondrial respiratory function [108]. Namisaki et al. also reported losartan as a potential drug for treating NASH due to its ability to suppress liver fibrosis in rats [109]. Furthermore, telmisartan was reported to prevent hepatocarcinogenesis, probably by inhibiting angiogenesis in cirrhosis [110].

In patients with chronic heart failure and animal models of AF, RAAS blockers attenuated atrial fibrosis and decreased the incidence of AF [111, 112]. Angiotensin converting enzyme inhibitors, like enalapril or cilazapril, reduce the duration of AF in canines with heart failure or pacing-induction via attenuating atrial fibrosis [113–116].

## CONCLUSIONS

NAFLD and AF are common chronic non-infectious diseases with rising incidences. NAFLD is an independent risk factor for AF. The pathogenesis includes decreased adiponectin levels, IR, activation of RAAS, and other common pathways like inflammation, oxidative stress, and fibrosis (Figure 1). While adiponectin can improve NAFLD, higher adiponectin concentrations may correlate with a greater incidence of AF. Adiponectin level does not have a linear relationship with the incidence of AF, a U-shape relationship.

IR is another important factor in the pathogenesis of NAFLD and AF. NAFLD is closely associated with IR in “Obese/Metabolic NAFLD” patients but not in genetic variants NAFLD patients. Moreover, IR increases the occurrence of AF involved in myocardial structural and electrical remodeling, and autonomic neuropathy.

In addition, RAAS contributes to the pathogenesis of NAFLD and AF significantly. The AT1 receptor may play a key role in the signal pathway. Nevertheless, statins and RAAS blockers have the capacity to improve both NAFLD and AF. More research is needed to identify common therapeutic targets for AF and NAFLD.
